# Synergistic Approach of High-Precision 3D Printing and Low Cell Adhesion for Enhanced Self-Assembled Spheroid Formation

**DOI:** 10.3390/bios15010007

**Published:** 2024-12-26

**Authors:** Chunxiang Lu, Aoxiang Jin, Chuang Gao, Hao Qiao, Huazhen Liu, Yi Zhang, Wenbin Sun, Shih-Mo Yang, Yuanyuan Liu

**Affiliations:** 1 School of Mechatronic Engineering and Automation, Shanghai University, Shanghai 200444, China; cxlu@shu.edu.cn (C.L.); 2845879177@shu.edu.cn (A.J.); gaochuang128634@163.com (C.G.); 1787758318@shu.edu.cn (H.Q.); zhangyishu@shu.edu.cn (Y.Z.); sunwenbin0516@126.com (W.S.); smyang@shu.edu.cn (S.-M.Y.); 2 School of Medicine, Shanghai University, Shanghai 200444, China; lesyinz@163.com; 3 National Center for Translational Medicine (Shanghai) SHU Branch, Shanghai 200444, China; 4 Wenzhou Institute of Shanghai University, Wenzhou 325000, China

**Keywords:** biofabrication, 3D printing, chip, spheroid

## Abstract

Spheroids, as three-dimensional (3D) cell aggregates, can be prepared using various methods, including hanging drops, microwells, microfluidics, magnetic manipulation, and bioreactors. However, current spheroid manufacturing techniques face challenges such as complex workflows, the need for specialized personnel, and poor batch reproducibility. In this study, we designed a support-free, 3D-printed microwell chip and developed a compatible low-cell-adhesion process. Through simulation and experimental validation, we rapidly optimized microwell size and the coating process. We successfully formed three types of spheroids—human immortalized epidermal cells (HaCaTs), umbilical cord mesenchymal stem cells (UC-MSCs), and human osteosarcoma cells (MG63s)—on the chip. Fluorescent viability staining confirmed the biocompatibility and reliability of the chip. Finally, drug response experiments were conducted using the chip. Compared to traditional methods, our proposed strategy enables high-throughput production of size-controlled spheroids with excellent shape retention, while enhanced gas exchange during culture improves differentiation marker expression. This platform provides an efficient and cost-effective solution for biosensing applications, such as drug screening, disease modeling, and personalized therapy monitoring. Furthermore, the chip shows significant potential for real-time in vitro monitoring of cellular viability, reaction kinetics, and drug sensitivity, offering valuable advancements in biosensor technology for life sciences and medical applications.

## 1. Introduction

In order to mimic natural three-dimensional (3D) structures, researchers have begun to focus on the study of cell aggregates. Spheroids, resembling spherical structures, are closer to the natural environment of cells within organisms due to this naturally occurring 3D structure [1,2]. Spheroids have a wide range of applications in various fields, such as bioengineering [3,4], histology, pharmacology [5,6,7], and oncology [8,9]. Particularly, they are extensively used in vitro to simulate tissue or tumor characteristics. When cancer cells spontaneously aggregate to form 3D structures resembling tumor spheroids, studies on cell proliferation, invasion, and treatment response can be conducted [10]. Studying the behavior of tumor cells in spheroids can provide better insights into the mechanisms of tumor occurrence and development [8,11,12]. Moreover, platforms for the fabrication and testing of spheroids are widely used in high-throughput preparation [13,14,15], drug screening [16,17,18], and spheroid communication studies [19]. Therefore, the development of cost-effective, reliable, and high-throughput spheroid experimental platforms is essential.

Researchers have been devoted to various methods for fabricating spheroids, including hanging drops, microwells, microfluidics, magnetic manipulation, and bioreactors [20,21,22]. The hanging drop method is cost-effective, where 10–30 μL of cell suspension is quantitatively aspirated and injected onto a culture plate. The culture plate is inverted to allow droplets to form spheroids under gravity. Tung et al. developed a robot-assisted hanging droplet culture and studied the appropriate microwell size by customizing a 384-well plate to create spheroids in a high-throughput manner [23,24]. Sun et al. developed a superhydrophobic chip-based hanging drop method for spheroid fabrication, with a designed medium reservoir overcoming the drawback of traditional hanging drop methods for long-term spheroid culture [25]. The microwell method confines cells within specified micrometer-sized pits under gravity, facilitating spheroid formation [26,27]. Chen et al. fabricated a high-throughput spheroid formation platform consisting of an array of 1024 non-adherent microwells. Spheroids could be reliably produced within the core area of a 2 cm × 2 cm chip [13]. Fukuda et al. synthesized photocrosslinkable chitosan and fabricated hydrogel microstructures via micromolding. Spheroid microarrays were prepared alone or cocultured on hydrogel microstructures [28].

The hanging drop method provides sufficient oxygen to spheroids precipitated at the bottom of the droplets, which is beneficial for maintaining spheroid viability and function. However, it results in cumbersome media exchange processes and a high rate of spheroid loss. In comparison, the microwell method does not require individually dispensing cell suspensions, reducing labor intensity, offering high throughput, and allowing for controllable sizes. However, the spheroids are located at the bottom of the device, leading to a slightly insufficient gas supply, which is disadvantageous for cell samples with high oxygen demands. The current traditional methods for preparing spheroid chips still rely on replica molding of polydimethylsiloxane (PDMS), which involves photolithography and other processing techniques [29]. This approach has drawbacks such as high equipment requirements, specialized skills, and significant labor intensity. Therefore, there is still a need for a low-cost, highly reproducible spheroid experimental device that combines the advantages of the hanging drop method and the microwell method.

In this work, we proposed a process strategy for establishing low-cell-adhesion conditions on a high-precision 3D-printed microwell chip, enabling the formation of multiple types of self-assembled spheroids. We studied the impact of printing angles on molding precision under support-free conditions, identifying the optimal manufacturing angle to produce high-precision and stable microwells. We validated the stamp-transfer method for applying PDMS coatings, specifically observing the morphology of the coated pore walls. Using internal fluid simulations and experiments, we modeled the maximum liquid pressure the chip could withstand with different microwell sizes and low-adhesion coating strategies, comparing these results with actual outcomes. This confirmed the reliability of the simulation model and helped determine the optimal microwell size. Through efficient and stable seeding, we successfully formed three types of spheroids—human immortalized epidermal cells (HaCaTs), umbilical cord mesenchymal stem cells (UC-MSCs), and human osteosarcoma cells (MG63s)—at various seeding densities. By controlling gas exchange, we observed a more pronounced expression of differentiation markers in HaCaTs compared to traditional spheroid culture methods. The proposed strategy utilizes the medium supply provided through channels above the microwells, enhancing nutrient transfer and metabolic waste removal. The through-hole design ensured sufficient oxygen supply and compatibility with real-time monitoring. This approach combines the minimal external matrix interference and high atmospheric supply advantages of the hanging drop method with the high-throughput efficiency of microwell-based methods, offering a streamlined and effective one-step distribution process.

## 2. Materials and Methods

### 2.1. Design of the Microwell Chip

**Figure 1:**
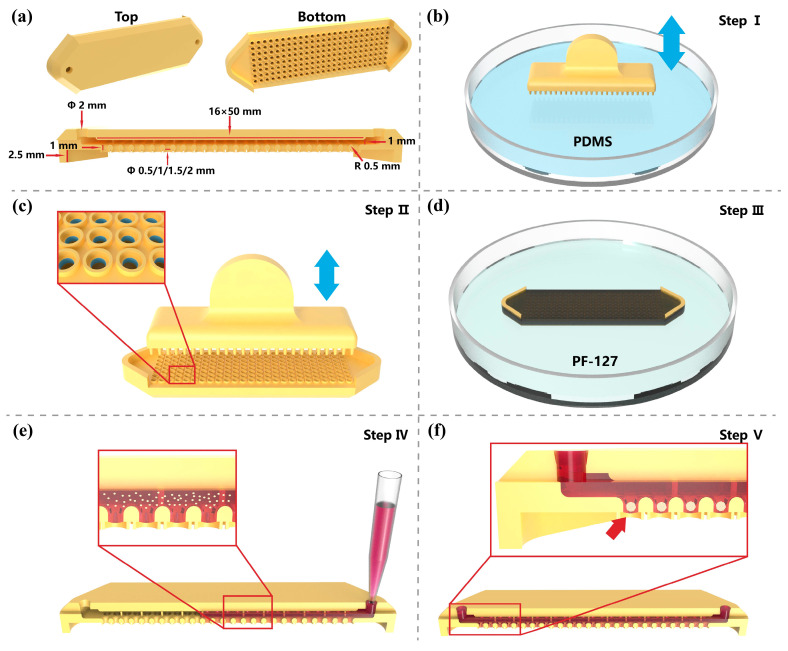
The fabrication process and operational procedure of the microwell chip. (**a**) Design of the microwell chip. (**b**) Dipping in PDMS. (**c**) Transferring PDMS in the microwell. (**d**) Dipping the stamp in PDMS. (**e**) Manual seeding of cells into the chip. (**f**) Spheroid formation in microwells.

Figure 1 outlines the fabrication and operation procedure of the chip. The 3D views of the chip are shown in Figure 1a. The chip features a channel with a height of 1 mm and through-holes with a depth of 1 mm. The through-holes are arranged in an array at the bottom of the chip. The diameter of the inlet/outlet channels is Φ 2 mm. The width of the chamber area channels is 16 mm, and the length is 50 mm. We designed four different microwell diameters: Φ 0.5 mm, Φ 1 mm, Φ 1.5 mm, and Φ 2 mm. The through-holes of the chip have an R 0.5 mm fillet at the top, which facilitates the even distribution of cells into the microwells and prevents cell sedimentation in the channel. The supports at both ends of the chip have a height of 2.5 mm to prevent the bottom of the chip from directly contacting the surface of the culture dish. Inside the microwells of the chip, we aim to form spheroids with diameters ranging from 200 μm to 500 μm, corresponding to the spheroid sizes recommended in most previous studies, which can prevent the formation of necrotic cores [20,30].

### 2.2. Fabrication of the Microwell Chip

Printing was performed using a Form 3B+ desktop 3D printer (Formlabs, Chicago, MI, USA) with surgical guide (SG) resin, selected for its biocompatibility. Post-printing, both the chip and coating stamp were subjected to ultrasonic cleaning in an isopropanol (IPA) solution for 20 min. Subsequently, they were immersed in anhydrous ethanol for 20 s to remove any residual IPA and resin. After cleaning, the chip was dried using a fan and baked at 60 °C for 30 min to ensure complete evaporation of the solvents. Finally, the chip and coating stamp were cured under 405 nm UV light for 30 min to enhance stability and ensure complete polymerization.

### 2.3. Coating of the Microwell Chip

The PDMS pre-polymer and cross-linker (Sylgard 184, Dow Corning, Midland, MI, USA) were mixed at a weight ratio of 10:1. A total of 5 g of the mixture was poured into a 10 cm diameter culture dish, evenly covering the bottom surface. The mixture was degassed under vacuum for 20 min to eliminate air bubbles, resulting in a uniform PDMS layer approximately 1 mm in height. The stamp was placed on the bottom of the dish, ensuring uniform adhesion of the PDMS to the stamp’s cylindrical structures. The PDMS-coated stamp was then transferred to the bottom of the chip, positioning the microwell side upward. Each cylinder was aligned with a microwell to facilitate the transfer of PDMS to the microwell walls. A fan was used to evenly direct airflow over the microwells, promoting uniform PDMS adhesion along the interior walls of the microwells. The chip was subsequently placed in a 70 °C oven for 3 h to fully cure the PDMS. The chips with PDMS coating were sterilized. The 75% ethanol was injected into the chip for 30 min of UV sterilization and then extracted. After rinsing three times with phosphate buffered saline (PBS). A 1.5% (*w*/*w*) solution of Pluronic F-127 (PF-127, Sigma-Aldrich, St. Louis, MA, USA) was prepared, and the chip was immersed in the filtered solution for 2 h. After immersion, the PF-127 solution was aspirated from the chip channels, and the chip was placed in an incubator to dry completely.

### 2.4. Contact Angle

The water contact angles on the resin surface under four conditions (no coating, PDMS coating, PF-127 coating, and PDMS-added PF-127 coating) were measured at room temperature via sessile drop contact angle measurement (n ≥ 3). Before the test, samples were adhered to a glass plate. Droplets of 10 µL water were dispensed onto the four surfaces, and contact angles were calculated using Image J software (Fiji, v 2.14.0).

### 2.5. Fluid Modeling

A finite element model was developed using COMSOL Multiphysics (v 6.2) to simulate fluid movement and critical leakage conditions inside the chip. The model was based on the Navier–Stokes equations, implemented through the “Laminar Flow” and “Phase Field” interfaces within the Multiphysics setup. A two-dimensional (2D) simplified model of the chip was obtained from a semi-sectional view of the 3D chip model. All simulated chip model cross-sectional dimensions were identical to those described in Section 2.1. Based on the experimentally measured water contact angles of the four coatings, we adjusted the contact angle data of the through-hole inner wall and the through-hole diameter in the simulation model to test the fluid behavior within the chip under different injection pressure ranges.

### 2.6. Cell Culture

#### 2.6.1. Cell Preparation

In the experiment, HaCaTs were purchased from ATCC (Manassas, VA, USA). MG63s were purchased from OriCell^®^ (Pudong, Shanghai, China). UC-MSCs were provided by the Shownin Biotechnologies Co., Ltd. (RC02003, Hefei, China). The cells were seeded in culture dishes and maintained in a CO_2_ incubator at 37 °C in a humidified atmosphere containing 95% air and 5% CO_2_. The cell concentration was maintained at 1 × 10^6^ cells mL^−1^. HaCaTs and MG63s were cultured in Dulbecco’s modified Eagle’s medium (DMEM) supplemented with 10% heat-inactivated fetal bovine serum (FBS), 100 U mL^−1^ penicillin, and 100 μg mL^−1^ streptomycin. UC-MSCs were cultured in a basic medium (BM) comprising a Minimum Essential Medium-alpha (MEM-*α*, Basalmedia, Shanghai, China). When the cells reached 80% confluence, they were detached using trypsin digestion and passaged at a ratio of 1:3.

#### 2.6.2. Cell Seeding and Spheroid Formation

The cells were digested with trypsin and diluted in DMEM/BM containing 10% FBS and 1% penicillin/streptomycin. Three different seeding densities were used: 5 × 10^5^ cells mL^−1^, 1 × 10^6^ cells mL^−1^, and 2 × 10^6^ cells mL^−1^. Approximately 1 mL of cell suspension was filled into the chip. After cell seeding, the microwell chip was placed in a culture dish containing 5 mL of PBS and covered with a lid. The culture dish was placed in a humidified atmosphere at 37 °C with 95% air and 5% CO_2_. Cell growth was measured every 2 days.

#### 2.6.3. Fluorescence Assay

To perform staining and imaging, the DMEM/BM inside the chip was aspirated. The viability of the cells was assessed using a live/dead cell staining kit (Solarbio). The 2 mM Calcein acetoxymethyl ester (Calcein-AM) (1.5 μL) and 1.5 mM Propidium iodide (PI) (4 μL) were separately dissolved in 1mL PBS to prepare the experimental solution. Calcein-AM staining solution was injected into the chip and then incubated in the dark at 37 °C for 20 min. After aspirating the staining solution, PBS was injected into the chip, followed by gentle shaking, and then aspirated. This PBS washing step was repeated twice. The PI staining solution was injected into the chip and incubated in a light-avoiding environment at room temperature for 5 min. After staining, the stained spheroids were washed twice with PBS. A fluorescence microscope (Ti2-d-pd, Nikon, Tokyo, Japan) was used for bright-field and stained spheroid observation, where live cells emitted green fluorescence and dead cells emitted red fluorescence.

#### 2.6.4. Histological and Immunofluorescence Analysis

To analyze the histology of HaCaTs spheroids, spheroids were harvested after 7 days of culture and gently washed with PBS. The spheroids were then fixed at 4 °C in 4% paraformaldehyde (PFA, Solarbio, Beijing, China) and embedded in optimum cutting temperature media. Frozen sections, 8 μm thick, were cut using a cryostat (n = 3). Immunofluorescence staining (n = 3) was performed to assess epidermal differentiation. Frozen tissue sections were thawed at room temperature, permeabilized for 1 h in a solution containing 0.1% Triton X-100 (diluted in PBS, Beyotime, Shanghai, China), and blocked for 1 h in PBS containing 1% bovine serum albumin (BSA, Beyotime) to prevent nonspecific binding. The samples from 7-day cultures were then incubated overnight at 4 °C with primary antibodies, rabbit anti-human cytokeratin 10, and cytokeratin 14 (CK10, CK14, diluted 1:500, Abcam, Cambridge, UK). After washing three times with PBS, the samples were incubated with secondary antibodies—goat anti-rabbit IgG conjugated to Alexa Fluor 488 and 594 (diluted 1:1000, Invitrogen, Carlsbad, CA, USA)—for 2 h at room temperature. Following additional PBS washes, the nuclei were counterstained with 4′,6-diamidino-2-phenylindole (DAPI, Beyotime) for 10 min at room temperature in the dark. Fluorescence microscopy was employed to observe and quantify the distribution and expression of the specific antibodies.

#### 2.6.5. Spheroid Cytotoxicity Assay

The drug response of MG63 cells was assessed in both 3D spheroid and 2D cultures. Prior to drug testing, spheroids formed from MG63 cells were cultured for 72 h, while cells in the 2D culture were incubated for 24 h before drug treatment. Doxorubicin (Dox, Solarbio) was dissolved in PBS and subsequently diluted in DMEM containing 15% FBS and 1% PS, with serial 10-fold dilutions prepared from an initial concentration of 1 mM. Varying concentrations of Dox were then added to MG63 cell cultures. After drug addition, both 2D and 3D cultures were incubated for 24 h. Following this incubation, cells were stained and imaged as described in the viability assay section. Cell viability of MG63 cells on different samples was assessed using a Cell Counting Kit-8 (CCK-8, Dojindo, Kumamoto, Japan). A 100 μL solution was drawn from each sample and transferred to a 96-well plate (Corning, New York, NY, USA). The plate was then placed in the measurement region of a microplate reader (Tecan, Männedorf, Switzerland) to record optical density (OD) at an absorbance wavelength of 450 nm (n = 3).

### 2.7. Image Analysis

The surface morphology of microwells was determined using scanning electron microscopy (SEM, ZEISS, Oberkochen, Germany) at an accelerating voltage of 5 kV. Before observing by SEM, the samples were sprayed with a 10 nm layer of Au (EM ACE600, Leica, Wetzlar, Germany). Using optical/fluorescence microscopy (Ti2-d-pd, Nikon), photographs of the microwells and spheroids were captured to measure the individual microwell and spheroid sizes. Image J software was employed to measure the horizontal and vertical axes and to calculate the average of these values (n ≥ 10). For the spheroid morphology, circularity and solidity were quantitatively analyzed based on 2D images. To estimate the viability of the spheroids cultured over a period of time, fluorescence images were obtained from the green and red fluorescence channels using Image J software. Live and dead cells were counted within the generated spheroid range to determine the ratio.

### 2.8. Statistical Analysis

Origin 2022 (OriginLab) software was used for all statistical analyses. All results are expressed as the mean ± standard deviation (s.d.), and error bars represent s.d. All experiments have been conducted with n ≥ 3. * *p* < 0.05 and ** *p* < 0.01 were considered statistically significant.

## 3. Results and Discussion

### 3.1. Formation Quality of the Microwell Chip

To promote the self-assembly of cells into spheroids through microwell culture, the microwell chip needs to meet the following requirements: (1) Even distribution to each microwell with a single injection. (2) The structure of the chip is designed to be integrated from bottom to top without the need for additional support. (3) Cells inside the microwells can be directly observed under a microscope. We designed an upper-level common channel and lower-level through-holes. This allows the cell suspension to be injected from the common channels directly into the lower-level through-holes. Additive manufacturing typically requires support structures to prevent collapse due to overhangs with angles less than 45°. Removing these supports after printing can damage the part, especially for non-professionals. The microwell chip avoids the need for support, greatly reducing labor intensity. Additionally, parts printed using stereo lithography appearance (SLA) often exhibit unavoidable layer lines due to the layer-by-layer manufacturing process, affecting surface roughness and optical transparency. We designed the microwells as through-holes, utilizing the adhesion of the material surface to the liquid, making the microwells easy to observe without liquid overflow.

**Figure 2:**
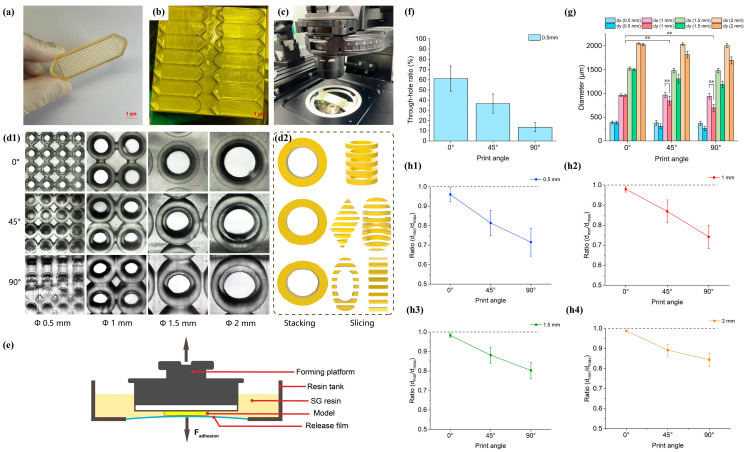
Formation and analysis of microwells. (**a**) The image of the microwell chip. (**b**) The microwell chip printed in batch without the need for additional support materials. (**c**) The chip holder ensures microwells are within the focal range of the microscope. (**d1**) Surface of the microwells observed under a microscope. Scale bar: 200 μm. (**d2**) Schematic diagram of layer stacking. (**e**) Schematic diagram of the adhesive force (F_adhesive_) exerted by the release film on the printed model. (**f**) The achievement rate of Φ 0.5 mm through-holes. (**g**) The specific formed sizes of microwells. (The dx, dy refers to the length measured in the *x*-axis/*y*-axis direction.) Asterisks indicate significant differences at ** *p* < 0.01. (**h1**–**h4**) The circularity at different sizes (the ratio of d_min_ to d_max_, where d_min/max_ is the minimum/maximum value of dx and dy within the same hole).

The first step in developing the microwell chip was to design appropriate sizes and verify the dimensional accuracy. Since additive manufacturing is a bottom-up process, it was necessary to examine the influence of different angles on the formation of microwells. We first validated the sizes of microwell formed at printing angles of 0°, 45°, and 90° for four diameters: Φ 0.5 mm, Φ 1 mm, Φ 1.5 mm, and Φ 2 mm. Characterization was performed under a microscope after cleaning and post-curing, as shown in Figure 2 for comparative analysis.

As shown in Figure 2(d1), the same model exhibited differences in the actual formation under three printing angles. Overall, the microwells formed well in the chip printed at a 0° angle. Regarding the through-hole rate, except for the Φ 0.5 mm microwell, all other microwells achieved a 100% through-hole rate. As depicted in Figure 2f, the Φ 0.5 mm microwell achieved the highest through-hole rate at the 0° printing angle. This is due to the Φ 0.5 mm microwell approaching the precision limit of the printer. By measuring the dx (diameter in the *x*-axis direction) and dy (diameter in the *y*-axis direction) of each microwell, significant differences in dx and dy were observed at 45° and 90° printing angles (Figure 2g). Among the three different printing angles, the variability in dy was noticeable. Analyzing the roundness at different printing angles, as shown in Figure 2(h1–h4), revealed that the ratio was closest to 1 with minimal deviation at the 0° printing angle. The ratio significantly decreased at 45° and 90° printing angles. Additionally, at the same printing angle, the larger the microwell, the smaller the deviation between the theoretical and actual values. Based on these results, we analyzed that this was due to the adhesion force (F_adhesion_) in the *z*-axis direction of the platform during SLA printing (Figure 2e), leading to cumulative errors through successive layers (Figure 2(d2)). Consequently, we chose the 0° angle as the optimal printing angle and eliminated the Φ 0.5 mm microwell due to its lower success rate.

### 3.2. Coating of the Microwell

**Figure 3:**
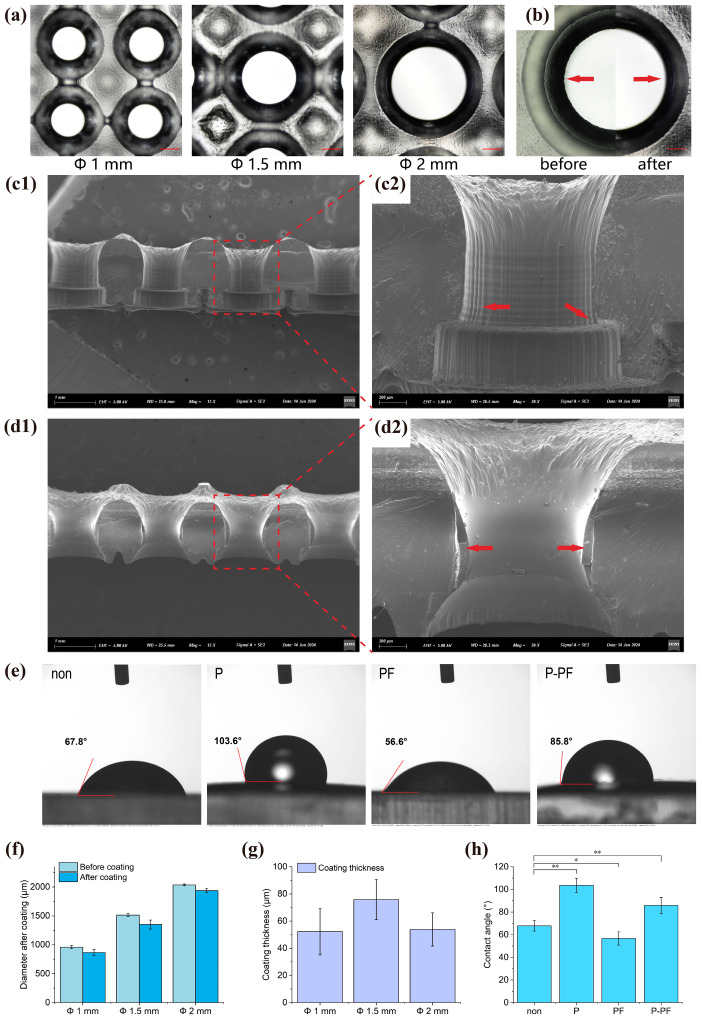
Morphological characterization of microwell coatings. (**a**) Microscopic images of microwells after PDMS coating. Scale bar: 200 µm. (**b**) Comparison of microwell wall surfaces before and after PDMS coating, with the left arrow showing the rough surface before coating and the right arrow showing the smooth surface after coating. Scale bar: 200 µm. (**c1**,**c2**) SEM observation of the cross-section (15× magnification) and local enlarged view (50× magnification) of the uncoated Φ 1 mm microwell. Arrows indicate layers and burrs. (**d1**,**d2**) SEM observation of the cross-section (15× magnification) and local enlarged view (50× magnification) of the Φ 1 mm microwell coated with PDMS. Arrows indicate the coating. (**e**) Water contact angles of no coating (non), PDMS coating (P), PF-127 coated (PF), and PDMS added PF-127 coating (P-PF) surfaces. (**f**) Diameter of microwells before and after PDMS coating. (**g**) Thickness of PDMS coating. (**h**) Comparison of water contact angles on four different surfaces. Asterisks indicate significant differences at * *p* < 0.05, ** *p* < 0.01.

**Figure 4:**
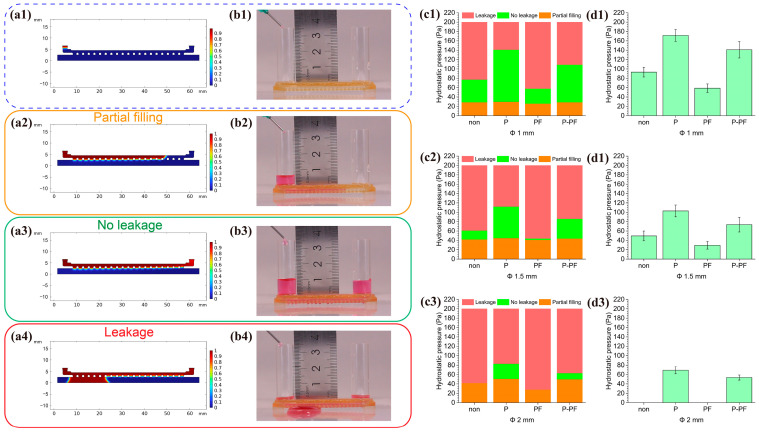
Simulation and experimental results of fluid behavior inside the chip. (**a1**–**a4**) Simulated 2D fluid behavior and leakage in the chip. (**b1**–**b4**) Fluid behavior in the 3D chip under continuous perfusion of the medium. (**c1**–**c3**) Specific numerical ranges of the three fluid outcomes in the simulated 2D chip. (**d1**–**d3**) Maximum hydrostatic pressure the 3D chip can withstand.

The morphology of the PDMS coating was observed under three different microwell sizes, as shown in Figure 3a. In bright-field observation, there was a noticeable difference in surface roughness before and after coating (Figure 3b). To further investigate this phenomenon, the microwells were cut open and their cross-sections were observed using SEM. PDMS was able to repair the layering lines and burrs generated during the printing process, resulting in smooth and uniform microwell wall surfaces after coating (as shown in Figure 3(c1,c2) and Figure 4(d1,d2)). The diameter after coating was measured, and the thickness of the PDMS coating was determined (Figure 3f,g). After stamp transfer, the PDMS coating demonstrated overall uniformity in the size of the microwells. PDMS adhered successfully to microwells of different sizes without any observed blockages, validating its feasibility.

Water contact angles were measured to compare the hydrophilicity under four conditions: no coating (non), PDMS coating (P), PF-127 coating (PF), and PDMS with added PF-127 coating (P-PF). As shown in Figure 3e,h, the water contact angle on the uncoated resin surface as the control group was 67.8°. After PDMS coating, the contact angle increased to 103.6°, indicating that PDMS significantly changed the resin surface to hydrophobic. When the resin was immersed in PF-127, the water contact angle decreased to 56.6°, indicating increased hydrophilicity. After PDMS coating followed by immersion in PF-127, the water contact angle was 85.8°. Compared to the uncoated surface, hydrophilicity decreased. In summary, there were significant differences in water contact angles under the four conditions. Hydrophilicity is an important indicator affecting the performance of internal fluid in the chip. This provides data support for establishing internal fluid simulations.

### 3.3. Internal Fluid Modeling of the Chip

The main challenge in forming hanging drops within through-holes is achieving the appropriate injection pressure. Insufficient injection pressure will result in the cell suspension failing to fill the chip, while excessive pressure can cause leakage. The different diameters and coatings of the microwell chip significantly influence the maximum hydrostatic pressure it can withstand. To demonstrate and simulate this process, two-dimensional models were developed to simulate fluid behavior within the chip under different microwell diameters and coatings, as shown in Figure 4(a1–a4). These are categorized into three outcomes: partial filling, no leakage, and leakage. By altering the contact angle of the inner microwell walls to simulate the four coating conditions, the injection pressures were summarized corresponding to each condition. As shown in Figure 4(c1–c3), simulation results revealed a strong correlation between the maximum hydrostatic pressure and the contact angle post-coating and microwell diameter. As the contact angle increases, so does the hydrostatic pressure tolerance. Structurally, the larger the microwell size, the lower the hydrostatic pressure tolerance. Specifically, for the Φ 2 mm microwell chip, neither the uncoated nor the PF-127 coated conditions could meet the requirement of complete liquid injection without leakage.

To validate the predictions of the simulation model, experiments were conducted. Figure 4(b1–b4) shows the fluid behavior within the 3D chip under continuous perfusion with the medium. By observing the maximum liquid level height, the corresponding hydrostatic pressure was calculated. The maximum hydrostatic pressures that various microwell diameters and coatings could withstand are plotted in Figure 4(d1–d3). The experimental results align reasonably well with the simulation predictions, confirming the reliability of the 2D simulation model. In the simulation, it was found that larger microwell sizes not only increased the difficulty of injection but also reduced the number of microwells per unit area, which in turn decreased the number of spheroids formed. Therefore, the Φ 1 mm microwell chip can accommodate higher spheroid throughput and a broader range of injection pressures, facilitating subsequent cell seeding and spheroid cultivation.

### 3.4. The Influence of Different Coatings on Spheroid Formation

To validate the ability of cells to form spheroids in microwell chips with different coatings, the proportion of spheroid formation reflects the differences in cell adhesion on different coatings. The microwell chip with a microwell diameter of Φ 1 mm was utilized to compare the spheroid formation proportions of four coatings (non, P, PF, P-PF). HaCaTs seeding density was maintained at 1 × 10^6^ cells mL^−1^.

**Figure 5:**
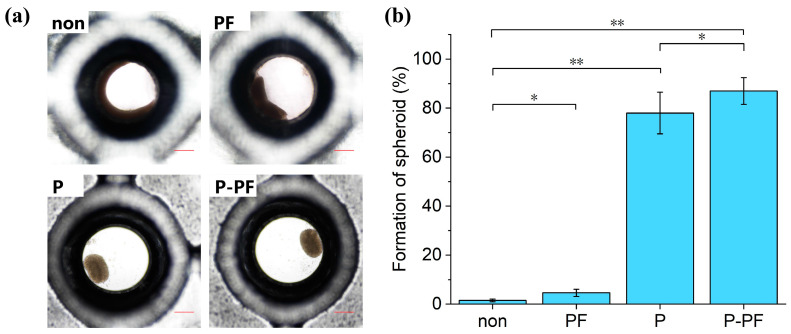
The influence of different coatings on spheroid formation. (**a**) The growth of cells inside the microwell chip with four coatings (non, PF, P, and P-PF) after 1 day. Scale bar: 200 µm. (**b**) The proportion of HaCaTs forming spheroids in the four groups of microwell chips. Asterisks indicate significant differences at * *p* < 0.05, ** *p* < 0.01.

After 1 day, observation under a microscope (Figure 5) revealed the spheroid formation. In the microwells coated with non and PF-127, most cells adhered to the surface with low spheroid formation. In contrast, microwells coated with PDMS and P-PF showed significant improvement, exhibiting higher spheroid formation proportion with well-rounded spheroids compared to the former coatings. The microwells coated with P-PF displayed a higher spheroid formation proportion than those coated solely with PDMS.

Pluronic is a non-ionic surfactant classified as an amphiphilic triblock copolymer, consisting of a central poly(ethylene oxide) block flanked by two poly(propylene oxide) blocks. Pluronic has been used to inhibit cell adhesion on PDMS surfaces [31,32]. However, SG resin coated with PF-127 still exhibited high cell adhesion. PDMS, known for its hydrophobicity and low cell adhesion properties, significantly enhanced spheroid formation and isolated the effects of SG resin. The experiments confirmed that the P-PF coating indeed reduces cell adhesion and achieves optimal spheroid formation proportion.

### 3.5. Integrated Biosensing Approaches for Spheroid

#### 3.5.1. Real-Time Spheroid Size and Viability Monitoring

We selected P-PF-coated Φ 1 mm microwell chips and validated them with several cell types, including HaCaTs, UC-MSCs, and MG63s. Cells were seeded at three densities: 5 × 10^5^ cells mL^−1^, 1 × 10^6^ cells mL^−1^, and 2 × 10^6^ cells mL^−1^. We measured spheroid size and viability at different cell seeding densities and conducted continuous cultivation for 7 days.

**Figure 6:**
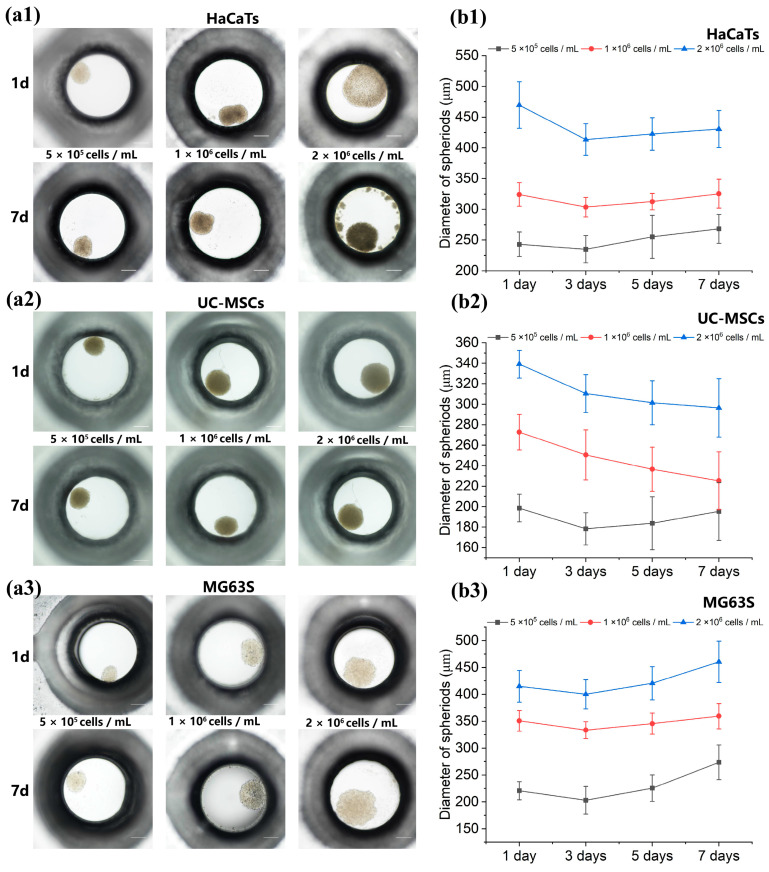
Fabrication and cultivation of spheroids on the microwell chips. (**a1**–**a3**) Bright-field images of HaCaTs, UC-MSCs, and MG63s spheroids within microwells after seeding with gradient cell densities on day 1 and day 7. Scale bar: 200 μm. (**b1**–**b3**) Diameters of HaCaTs, UC-MSCs, and MG63s spheroids measured on days 1, 3, 5, and 7.

Microscopic images of the spheroids are presented in Figure 6(a1–a3). By comparing the diameters of spheroids measured on days 1, 3, 5, and 7, it was observed that spheroid size was dependent on the initial cell seeding density and could be modulated by varying this parameter (Figure 6(b1–b3)). A reduction in spheroid diameter was noted on day 3 across all samples. While cells formed initial spheroids within 1 day, they exhibited loose cellular cohesion, which progressively tightened, resulting in the observed diameter reduction by day 3. Subsequently, diameters gradually increased by days 5 and 7, with MG63 spheroids displaying the most pronounced increase, whereas UC-MSC spheroids exhibited a continuous decrease in diameter. This behavior is consistent with previous findings [18,33,34].

**Figure 7:**
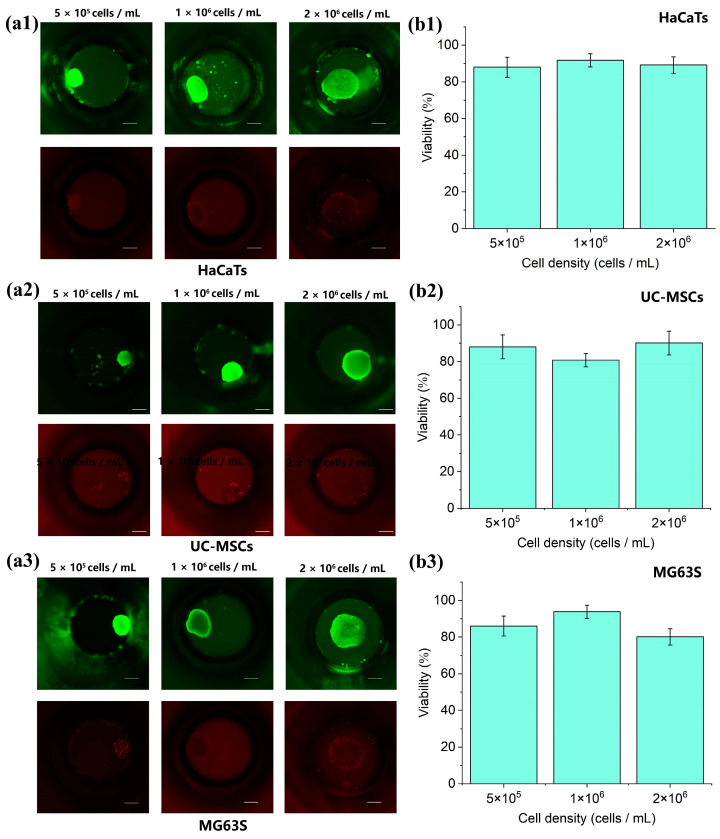
Viability of spheroids on the microwell chips. (**a1**–**a3**) Microscopic images of HaCaTs, UC-MSCs, and MG63s spheroids cultured continuously on the microwell chips for 7 days. Spheroids stained with Calcein-AM and PI. Scale bar: 200 μm. (**b1**–**b3**) Viability of HaCaTs, UC-MSCs, and MG63s spheroids on day 7 on the microwell chips.

Spheroid viability was assessed on day 7 using viability staining, performed under conditions of daily medium exchange (Figure 7(a1–a3)). Spheroids across all three initial seeding densities maintained viability rates exceeding 80% (Figure 7(b1–b3)), highlighting their potential for long-term cultivation.

#### 3.5.2. Morphological Analysis of Spheroid Shape Consistency

To compare the spheroid shapes formed by the hanging drop method and the microwell chip, we measured the circularity and sphericity of the three types of spheroids (HaCaTs, UC-MSCs, and MG63s) after three days. The concepts of circularity and sphericity indices were introduced to quantify the shape of the spheroids [35]. Area and perimeter refer to the respective area and perimeter of the analyzed spheroidal object. Sphericity ranges from 0 to 1, where values closer to 1 indicate a higher degree of spherical cross-section.

(1) $$Sphericity=\frac{\sqrt{4\pi \times Area}}{Perimeter}$$

(2) $$Circularity=\frac{4\pi \times Area}{{Perimeter}^{2}}$$

**Figure 8:**
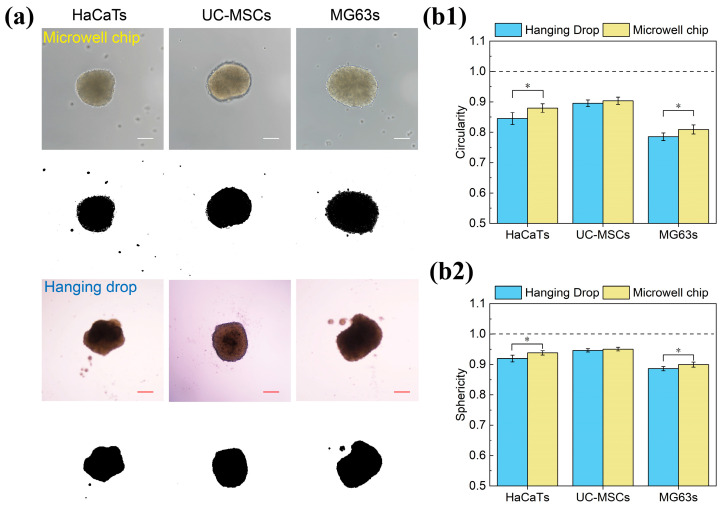
Measurement and analysis of the spherical shape of the spheroid. (**a**) Bright-field image of the spheroids, followed by binarization processing using ImageJ to evaluate shape and area. Scale bar: 100 µm. (**b1**,**b2**) Comparison of the circularity and sphericity of HaCaTs, UC-MSCs, and MG63s spheroids formed by the hanging drop method and the microwell chip method. Asterisks indicate significant differences at * *p* < 0.05.

Using bright-field images, the images were binarized with ImageJ (Figure 8a), and the area and perimeter were measured and plugged into the equations to calculate the circularity and sphericity indices (Figure 8(b1,b2)). We observed that, except for MG63s, most spheroids maintained a circularity above 0.8. The circularity of spheroids was also cell-type-dependent. Spheroids of UC-MSCs exhibited better circularity compared to MG63s and HaCaTs. The values of circularity and sphericity indices quantitatively demonstrate that spheroids cultured using the microwell chip method maintained a better spherical shape compared to the traditional hanging drop method. This suggests that the microwell chip method provides an improved environment for maintaining uniform spheroid morphology, which is crucial for reproducibility and functionality in further biological applications.

#### 3.5.3. Evaluation of Cell Differentiation and Fluorescence Signal Intensity

To evaluate the differentiation potential of spheroids formed using the microwell chip, a comparison was made between spheroids generated by the hanging drop and the microwell method. After 7 days of culture, immunofluorescence staining was performed on HaCaTs spheroids to assess epithelial differentiation markers, CK10 and CK14.

**Figure 9:**
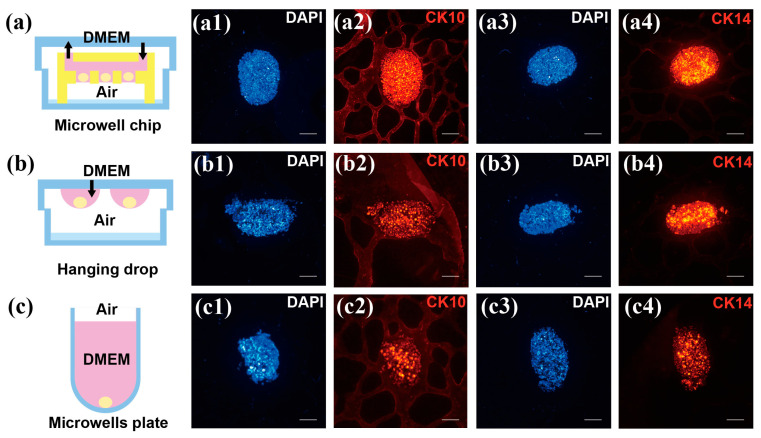
Immunofluorescence staining of HaCaTs spheroids. (**a**,**b**,**c**) Schematics of different culture methods. (**a1**,**b1**,**c1**) and (**a3**,**b3**,**c3**) show DAPI-stained images of spheroids after 7 days of culture. (**a2**,**b2**,**c2**) show CK10-stained images of spheroids after 7 days of culture. (**a4**,**b4**,**c4**) show CK14-stained images of spheroids after 7 days of culture. Scale bar: 100 μm.

Immunofluorescence microscopy, as shown in Figure 9, reveals the blue sections representing cell nuclei stained with DAPI. The red sections in Figure 9(a2–c2) indicate CK10 expression, an early differentiation marker of the epidermis primarily present in the spinous and granular layers [36,37]. CK14, shown in red (Figure 9(a4–c4)), is mainly expressed in the basal layer of stratified squamous epithelium, playing a key role in maintaining cell morphology and resistance to external mechanical stress [38,39,40].

The immunofluorescence staining indicated that HaCaTs spheroids differentiated with CK10 and CK14 markers under all three culture conditions. However, spheroids cultured using the U-shaped bottom microwell plate exhibited the weakest fluorescence intensity due to limited gas exchange. In contrast, spheroids cultured via the hanging drop method showed more pronounced differentiation due to stimulation from the air–liquid interface. Spheroids cultured in the microwell chip demonstrated the strongest fluorescence intensity, which can be attributed to the advantages of the microwell chip: it not only provides a high oxygen supply, similar to the hanging drop method, but also overcomes the limitations of nutrient exchange inherent. Improved media exchange promotes better metabolism and differentiation in epidermal cells.

#### 3.5.4. Spheroid-Based Drug Response Assessment

To demonstrate the drug testing capability of the microwell chip, we seeded MG63 cells at a density of 1 × 10^6^ cells mL^−1^ and cultured them in the chip for 72 h to form spheroids. The spheroids were then treated with doxorubicin (Dox), followed by staining with Calcein-AM and PI to assess live and dead cells.

**Figure 10:**
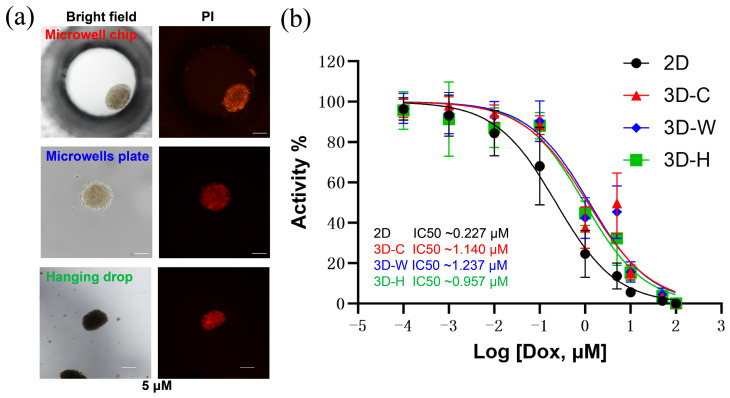
Drug response of spheroids. (**a**) Images of spheroids stained for dead cells after exposure to 5 μM of doxorubicin. Scale bar: 200 μm. (**b**) Drug–response curves of doxorubicin treatment for spheroids generated from 2D culture (2D), micropore chip (3D-C), U-shaped micropore plate (3D-W), and hanging drop method (3D-H).

The dose–response curves in Figure 10 demonstrate a clear dose-dependent impact of Dox on the viability of MG63 cells cultured in both 3D and 2D. As shown in Figure 10b, the IC50 values are 0.227 μM for 2D cultures, 1.140 μM for spheroids in the microwell chip, 1.237 μM for spheroids in the microwells plate, and 0.957 μM for those generated by the hanging drop method. MG63 cells grown as 3D spheroids displayed approximately 5-fold higher drug resistance compared to 2D monolayers. Notably, the IC50 values for 3D spheroids cultured in the microwell chip and microwell plate were similar, while the spheroids generated via the hanging drop method exhibited higher standard deviation. This suggests that generating spheroids of uniform size enhances the reliability and reproducibility of drug response data.

The drug response experiment demonstrated the potential of the microwell chip in facilitating drug screening applications. The chip’s design allows for real-time fluorescence observation in situ, eliminating the need to transfer spheroids to microtiter plates. The one-step injection and distribution process enhances efficiency and ease of use. In summary, we present a novel composite technique that could be further optimized by adjusting the medium composition and exploring external stimulation factors to advance spheroid biofunctionality. The fabrication strategy proposed in this study offers an efficient and cost-effective method for high-throughput spheroid production, facilitating biosensing applications such as drug screening, disease model development, and personalized therapeutic response monitoring.

## 4. Conclusions

In this study, we proposed a fabrication strategy that integrates high-precision 3D printing with low-cell-adhesion coatings. Using this approach, we successfully produced microwell chips with controllable dimensions and uniform coatings. The experimental results demonstrated that (1) spheroids were efficiently and stably formed from HaCaTs, UC-MSCs, and MG63s cells at varying seeding densities; (2) spheroids cultured on the microwell chip exhibited superior sphericity; (3) HaCaTs spheroids under high oxygen supply and media exchange conditions displayed enhanced expression of differentiation markers; and (4) the microwell chip facilitated drug screening assays. This approach has the potential to advance spheroid-based biosensing applications in drug screening and disease modeling, particularly with further optimization of the culture environment and incorporation of external stimuli to enhance biological functionality.

## Data Availability

Data are contained within the article and Supplementary Material.

## Supplementary Materials

The following supporting information can be downloaded at: https://www.mdpi.com/article/10.3390/bios15010007/s1, Figure S1: Preparation of microwell chip and coated transfer stamp. (a) Design of the transfer stamp. (b) Combination schematic diagram. (c) Transfer of PDMS using the stamp. (d) Actual combination situation; Figure S2: Electron microscope characterization of mi-crowell chip. (a1–c1) View of the microwells on the bottom of the chip. (a2–c2) View of microwells in the chip flow channel. (a3–c3) Microwell walls before coating. (a4–c4) Microwell walls after coating; Figure S3: Establishment of a 2D simulation model. (a) 2D model view. (b) Blue lines represent the coated variable wettability walls. (c) Simulation result for φ 1.5 mm microwells with PF coating. (d) Simulation result for φ 2 mm microwells with PF coating; Figure S4: Overview of the microwells. (a) Chip with PDMS coating. (b) Chip with PDMS and PF-127 coating. Scale bar: 5 mm.; Video S1: Video of medium injection into the internal channel of the chip and liquid exchange.
