# Supplementary material for: Synergistic Approach of High-Precision 3D Printing and Low Cell Adhesion for Enhanced Self-Assembled Spheroid Formation
Source: Biosensors (Basel). 2024 Dec 26;15(1):7. doi: 10.3390/bios15010007 (PMC11764235; doi:10.3390/bios15010007)
Supplement: Supplementary file 1 [file biosensors-15-00007-s001.zip › biosensors-3305653-supplementary.pdf]

Synergistic Approach of High-Precision 3D Printing and Low Cell Adhesion for Enhanced Self-Assembled Spheroid Formation

Chunxiang Lu[a], Aoxiang Jin[a], Chuang Gao[a], Hao Qiao[a], Huazhen Liu[d], Yi Zhang[a], Wenbin Sun[a], Shih-mo Yang[a], Yuanyuan Liu[a,b,c*]

a School of Mechatronic Engineering and Automation, Shanghai University, Shanghai, 200444, China

b National Center for Translational Medicine (Shanghai) SHU Branch, Shanghai, 200444, China

c Wenzhou Institute of Shanghai University, Wenzhou, 325000, China

d School of Medicine, Shanghai University, Shanghai 200444, China

* Correspondence: yuanyuan_liu@shu.edu.cn
Tel.: +86-15900611572

1. Preparation of microwell chip and coated transfer stamp

As shown in Supplementary Figure S1a, the design of the stamp includes an array of micropillars that align with the microwells of the microchip. In Supplementary Figure S1b, the combination of these two components allows the PDMS adhered to the micropillars to transfer onto the inner walls of the microwells. A slight gap is maintained between the micropillars and microwells to ensure that the PDMS is not completely isolated from the microwell walls. Figure S1c and S1d illustrate the actual process, demonstrating that the two parts can be assembled for effective coating transfer.

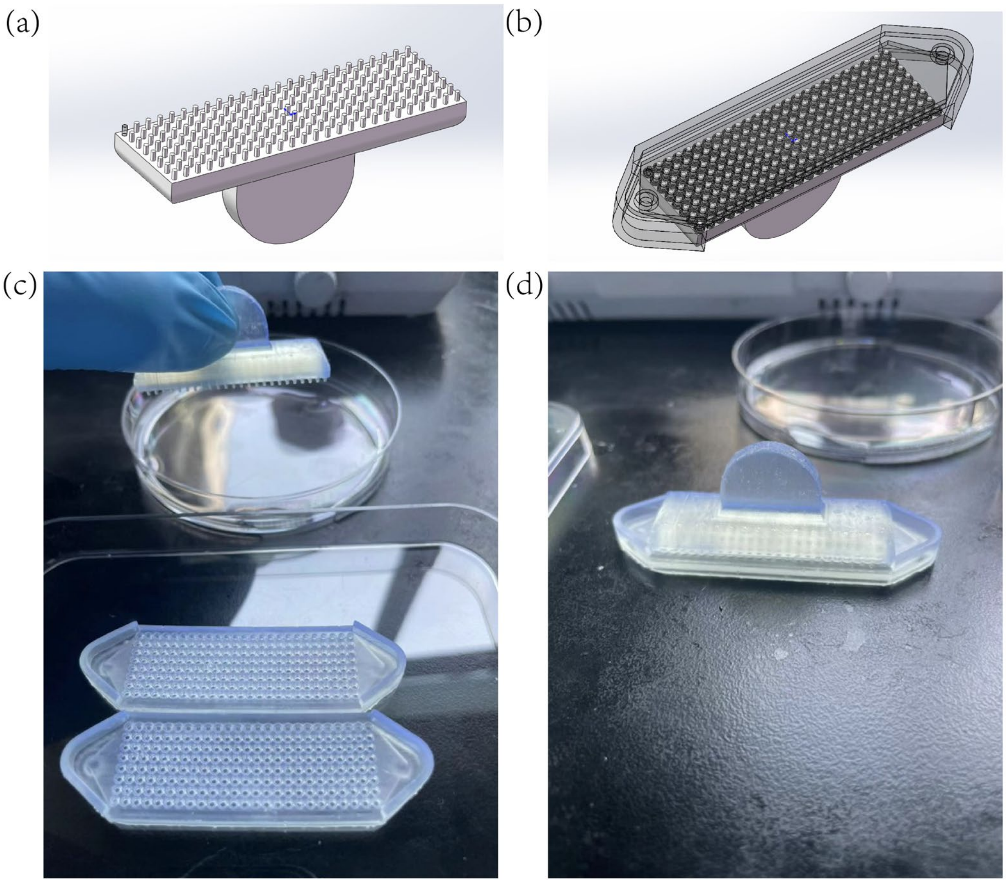

Figure S1 Preparation of microwell chip and coated transfer stamp. (**a**) Design of the transfer stamp. (**b**) Combination schematic diagram. (**c**) Transfer of PDMS using the stamp. (**d**) Actual combination situation.

2. Electron microscope characterization of microwell chip

To better observe the interior surface of the microwells after coating, the chip was cut to isolate the bottom array section, and three sizes of microwells were bisected for detailed examination. As shown in Figure S2, scanning electron microscopy (SEM) images revealed a clear PDMS coating on the inner walls of all three pore sizes. In comparison to the uncoated pores, which displayed a rough surface texture, the coated microwells had significantly smoother surfaces. This confirms that the PDMS coating transfer method using printed stamps is both feasible and reliable.

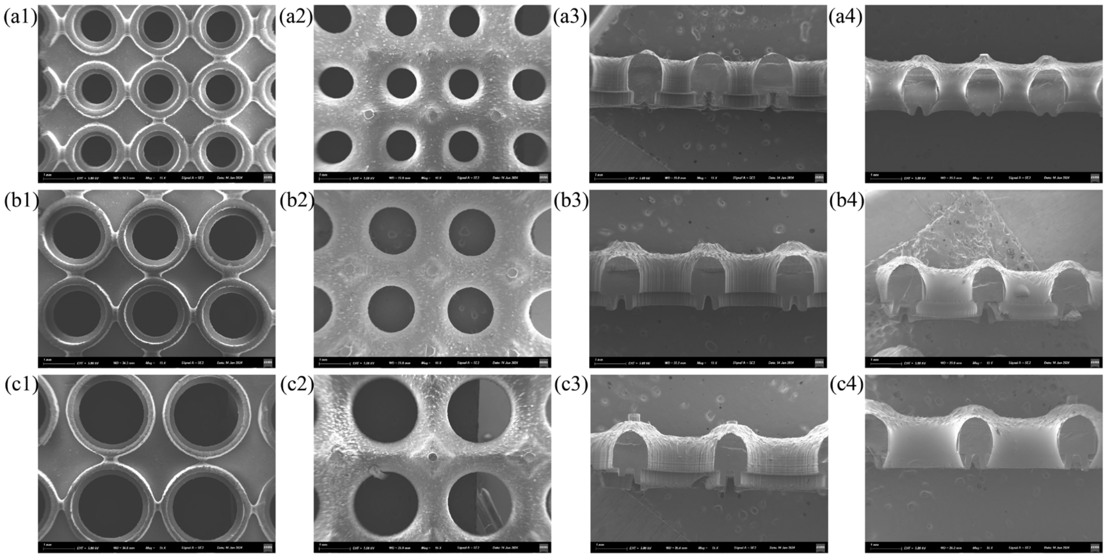

Figure S2 Electron microscope characterization of microwell chip. (**a1**,**c1**) View of the microwells on the bottom of the chip. (**a2**,**c2**) View of microwells in the chip flow channel. (**a3**,**c3**) Microwell walls before coating. (**a4**,**c4**) Microwell walls after coating.

3. Establishment of a Two-Dimensional Simulation Model

By converting a cross-sectional view of the 3D chip into a 2D simulation model, as shown in Figure S3a, two wettability wall conditions were established. In Figure S3b, the blue lines represent walls simulating the coated surfaces, while the remaining lines default to SG resin water contact angle data. By adjusting the water contact angle data corresponding to the coating, the leakage condition of the chip after liquid injection was simulated. The top left corner serves as the liquid inlet, with all other outlets connected to the atmosphere. By modifying the inlet pressure, critical liquid pressure values for different microwell sizes and coating conditions were validated. In Figure S3c, under the PF coating water contact angle data for a φ 1.5 mm microwell, leakage occurs when the inlet pressure is 50 Pa. In Figure S3d, with the PF coating water contact angle data for a φ 2 mm microwell, the channel remains unfilled at pressures below 28 Pa and exhibits immediate leakage above 28 Pa. Under these conditions, the chip does not meet liquid injection requirements, and this was also confirmed by experimental results.

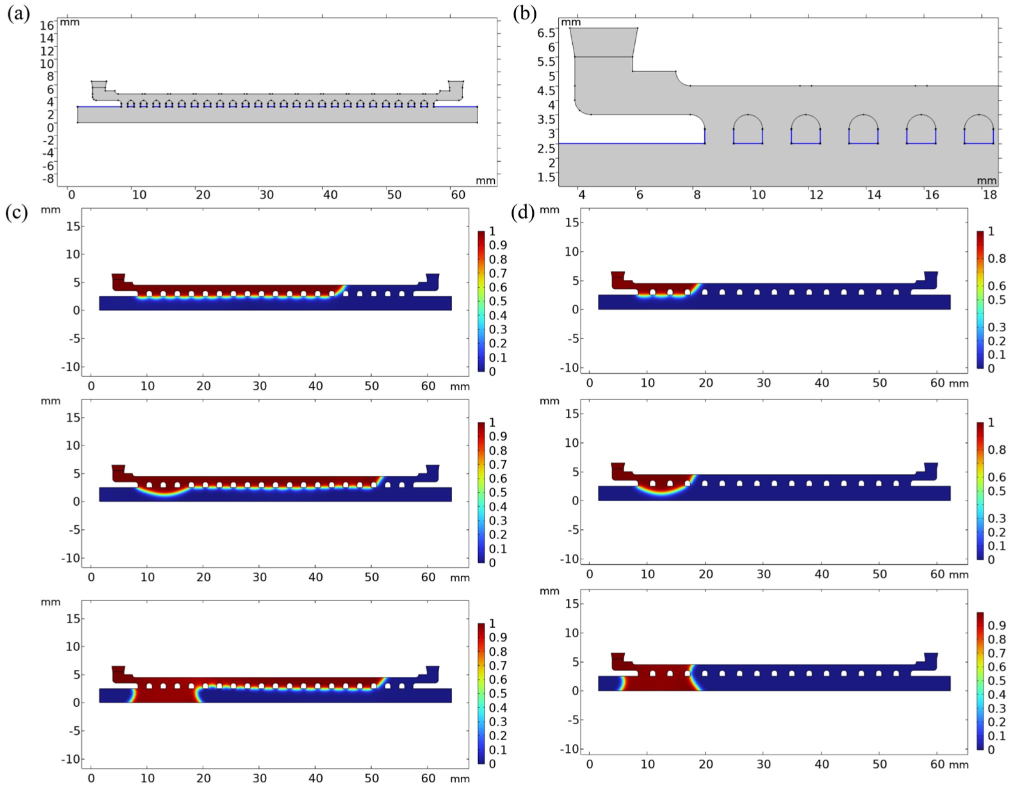

Figure S3 Establishment of a 2D simulation model. (**a**) 2D model view. (**b**) Blue lines represent the coated variable wettability walls. (**c**) Simulation result for φ 1.5 mm microwells with PF coating. (**d**) Simulation result for φ 2 mm microwells with PF coating.

4. Full view of microwells

To accommodate the limited field of view at 4x magnification, which only allows observation of four wells at a time, we utilized the continuous imaging function of the NIS microscope software to reconstruct the bright-field images of the entire microwell chip. Using a 12×8 array chip containing 96 wells, we observed the following results: Figure S4a represents the chip with only a PDMS coating, exhibiting a spheroid formation rate of 68/96, approximately 70.8%. Figure S4b represents the chip with a PDMS and PF-127 coating, which achieved a spheroid formation rate of 79/96, approximately 82.3%.

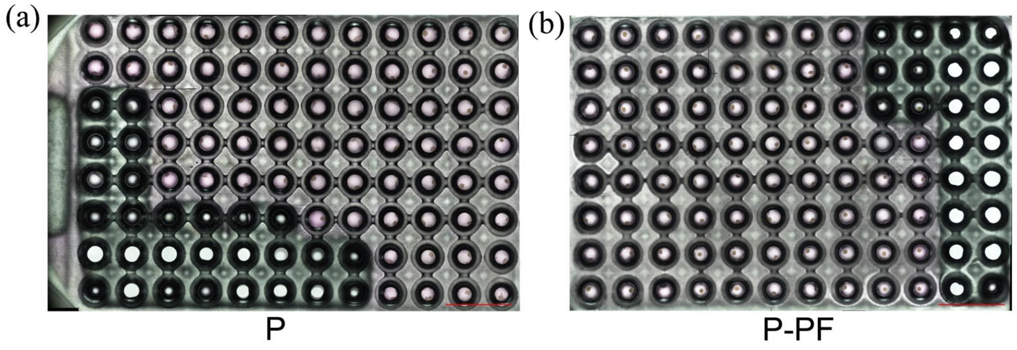

Figure S4 Overview of the microwells. (**a**) Chip with PDMS coating. (**b**) Chip with

PDMS and PF-127 coating. Scale bar: 5 mm.

5. Video of medium injection into the internal channel of the chip and liquid exchange

As demonstrated in Supplementary Video S1, a 1 mL pipette was used to inject a full 1 mL of suspension into the chip. After injection, no leakage was observed from the bottom of the chip, even during minor movements or handling. During the medium exchange, the liquid in the channel is removed, and less than 1 mL of fresh medium is introduced. In the video, although 1 mL was initially injected, the remaining medium in the pipette tip indicates that a portion of the medium remains in the bottom of the microwells during extraction. This results in the actual amount of extracted liquid being less than 1 mL; therefore, it is recommended to inject approximately 800 μL of fresh medium instead of the full 1 mL.
